# Cancer and the LGBTQ Population: Quantitative and Qualitative Results from an Oncology Providers’ Survey on Knowledge, Attitudes, and Practice Behaviors

**DOI:** 10.3390/jcm6100093

**Published:** 2017-10-07

**Authors:** Christina L. Tamargo, Gwendolyn P. Quinn, Julian A. Sanchez, Matthew B. Schabath

**Affiliations:** 1 Department of Health Outcomes and Behavior, H. Lee Moffitt Cancer Center and Research Institute, 12902 Magnolia Drive, Tampa, FL 33612, USA; christina.tamargo@med.miami.edu (C.L.T.); gwen.quinn@moffitt.org (G.P.Q.); 2 Department of Oncologic Sciences, Morsani College of Medicine, University of South Florida, 12901 Bruce B Downs Blvd, Tampa, FL 33612, USA; julian.sanchez@moffitt.org; 3 Department of Gastrointestinal Oncology, H. Lee Moffitt Cancer Center and Research Institute, 12902 Magnolia Drive, Tampa, FL 33612, USA; 4 Department of Cancer Epidemiology, H. Lee Moffitt Cancer Center and Research Institute, 12902 Magnolia Drive, Tampa, FL 33612, USA

**Keywords:** LGBTQ, sexual and gender minorities, providers, attitudes, behaviors, education, training

## Abstract

Background: Despite growing social acceptance, the LGBTQ population continues to face barriers to healthcare including fear of stigmatization by healthcare providers, and providers’ lack of knowledge about LGBTQ-specific health issues. This analysis focuses on the assessment of quantitative and qualitative responses from a subset of providers who identified as specialists that treat one or more of the seven cancers that may be disproportionate in LGBTQ patients. Methods: A 32-item web-based survey was emailed to 388 oncology providers at a single institution. The survey assessed: demographics, knowledge, attitudes, and practice behaviors. Results: Oncology providers specializing in seven cancer types had poor knowledge of LGBTQ-specific health needs, with fewer than half of the surveyed providers (49.5%) correctly answering knowledge questions. Most providers had overall positive attitudes toward LGBTQ patients, with 91.7% agreeing they would be comfortable treating this population, and would support education and/or training on LGBTQ-related cancer health issues. Conclusion: Results suggest that despite generally positive attitudes toward the LGBTQ population, oncology providers who treat cancer types most prevalent among the population, lack knowledge of their unique health issues. Knowledge and practice behaviors may improve with enhanced education and training on this population’s specific needs.

## 1. Introduction

The lesbian, gay, bisexual, transgender, and queer/questioning (LGBTQ) community, also referred to as sexual and gender minorities (SGM), is a medically underserved and understudied population in the United States [1,2]. The terms “lesbian”, “gay”, and “bisexual” typically refer to sexual attraction or sexual orientation; “transgender” refers to individuals who do not identify with their assigned sex at birth, as opposed to “cisgender”, in which an individual’s sex at birth aligns with his or her gender identity [3,4]; “queer” can refer to any and all groups in the LGBTQ spectrum; and “questioning” refers to the exploration of either sexual orientation or gender identity [2,5,6]. It is estimated that 3–12% of adults in the United States identify as LGBTQ [1,7]. Despite growing social acceptance, this population continues to face barriers to healthcare including difficulty obtaining and/or affording health insurance coverage, fear of stigmatization by healthcare providers, and healthcare providers’ lack of knowledge about LGBTQ-specific health issues [8]. Moreover, one in five transgender patients seeking healthcare are still turned away by providers [5,9,10]. In addition to facing systematic and provider-related barriers, LGBTQ persons experience a multitude of health disparities. For example, gay, lesbian, and bisexual adults and youth are at increased risk for depression, anxiety, suicide, and substance abuse; lesbian, and bisexual women are more likely to be obese than straight women [11,12,13,14].

In addition to these general mental and physical health disparities, the LGBTQ community also has increased risks for some cancers. A recent review of the literature [2] highlighted seven cancer sites that may disproportionately affect the LGBTQ population: anal, breast, cervical, colorectal, endometrial, lung, and prostate cancers. Briefly, anal cancer may be more prevalent among gay, bisexual, and other men who have sex with men for multiple reasons. First, human papillomavirus (HPV), a sexually transmitted infection (STI), is the primary cause of anal cancer [14,15]. Men who have sex with men (MSM) are at greater risk of HPV transmission due to anal sex practices [16]. In addition, HIV-infected persons are more likely to develop anal cancer, and gay and bisexual men are more likely to be HIV-infected [17,18]. While anal cancer disproportionately affects gay men, breast cancer may affect lesbian and bisexual women at greater rates than heterosexual women. There are limited data that explore these differences; however, lesbian and bisexual women more commonly experience a variety of breast cancer risk factors, including reduced pregnancy rates, smoking, and obesity [5,19]. Furthermore, some studies suggest lesbian and bisexual women are less likely to get mammography due to barriers to healthcare coverage and negative relationships with healthcare providers [20,21,22]. However, more research is needed to determine if these factors correspond to increased breast cancer risk [2]. HPV contributes not only to higher rates of anal cancer among MSM, but also to higher rates of cervical cancer among lesbian and bisexual women [2,23]. Recent studies have shown that STIs may be transmitted through sexual contact between women [24]; further, many women who have sex with women (WSW) have had sexual contact with men in the past [25]. Thus, it is likely for WSW to contract HPV, the leading cause of cervical cancer [23]. Similarly, there may be lower rates of Papanicolaou (Pap) testing among WSW because of the mistaken belief that they are not at risk for cervical cancer [2]. As such, the higher rates of cervical cancer among lesbian and bisexual women may be attributed, in part, to a misconception of HPV transmission resulting in the observed lower rates of Pap testing among this population. Colon and rectal cancers may disproportionately affect the LGBTQ populations due primarily to elevated risk factors, including smoking and obesity, within these groups [26,27,28]. However, further research is necessary to determine if there are differences in colorectal cancer rates between LGBTQ and majority populations [2]. Similar conclusions, or lack thereof, can be made regarding the relationship between LGBTQ status and lung cancer. While the prevalence of smoking, the leading cause of lung cancer, is higher in LGBTQ populations, a lack of research on LGBTQ and lung cancer makes it difficult to determine whether there is an association between the two [29,30]. However, a recent study showed that lung cancer incidence is greater among HIV-infected individuals than in the general population [31]; because HIV is more common in LGBTQ populations, specifically gay and bisexual men, this could highlight an additional distinction in lung cancer occurrence between sexual minority and heterosexual individuals [17]. A lack of published research also characterizes prostate cancer diagnosis in MSM. Unlike the other cancers described, no published data exist that compare risk factors for prostate cancer between gay, bisexual, and heterosexual men [2]. However, studies suggest that screening for prostate cancer with the prostate-specific antigen (PSA) test may be less common in men with HIV and/or some groups of MSM—for example, PSA testing among African American MSM was found to be lower than that of heterosexual African Americans [32,33].

To identify and address potential health disparities among LGBTQ populations, it is imperative to collect accurate sexual orientation and gender identity (SOGI) data which are often not collected in medical records and research databases. When SOGI data are not collected in the healthcare delivery setting, there may be a missed opportunity to discuss prevention, early detection, and screening for LGBTQ subgroups that may be at an increased risk for certain cancers. A lack of collection and nondisclosure can also result in a missed opportunity to discuss potential psychosocial issues specific to members of the LGBTQ population, as well as to make referrals to other health care professionals, such as social workers or psychologists, and community resources, which could have a positive impact on quality of life [34]. As SOGI data collection is currently a somewhat novel concept, it is imperative to develop training for providers and best practices for collection. Ultimately, when SOGI data are collected and subsequently applied in a meaningful and culturally sensitive manner, the quality of cancer care provided to the LGBTQ population can be improved [34].

Despite being an underserved population, few studies have explored healthcare concerns and needs of the LGBTQ population in regards to cancer. The Institute of Medicine also suggests few physicians are knowledgeable about and sensitive to LGBTQ-specific health issues [5]. In this current analysis, we sought to assess quantitative and qualitative responses from a subset of providers who identified as specialists that treat one of the aforementioned seven cancers [2] that may be disproportionate in LGBTQ patients.

## 2. Materials and Methods

### Study Population and Survey

The 33-item web-based survey was developed based on existing literature on LGBTQ health and healthcare experiences [3,35]. Details on the development and administration of the survey are reported elsewhere [35]. Briefly, the original sample included all Medical Doctors (MDs), Physician Assistants (PAs), and Nurse Practitioners (NPs) practicing at Moffitt Cancer Center (Tampa, FL, USA) using the Dillman Method [34]. In this study, we sought to examine those providers who specialized in one or more of the seven cancer sites (i.e., anal, breast, cervical, colorectal, endometrial, lung, and prostate cancers) that may disproportionally affect LGBTQ persons [2]. Chesapeake institutional review board (Columbia, MD, USA) approved the study and a waiver of documentation of written informed consent was provided.

The survey included five sections: demographics (eleven questions), knowledge (six questions), attitudes (five questions), practice (seven questions), and open comments (four questions). Three questions were removed for this analysis due to possible ambiguity in the way they were worded. The knowledge, attitudes, and practice behaviors sections included statements with the following response options: strongly disagree, disagree, neutral/don’t know, agree, and strongly agree.

The demographics section assessed gender, age group, sexual orientation, race, religious identity, licensure, year of graduation from professional school, specialty/clinic, average number of patients per week, and percentage of patients who have identified as LGBTQ. The knowledge section of the survey included six statements regarding LGBTQ patient-provider communication, risks of HPV and breast cancer among lesbians, screening tests for men who have sex with men (MSM), LGBTQ mental health issues, and transgender health insurance trends. The attitudes section consisted of five statements which assessed comfort in treating LGBTQ patients, belief of unique health needs, desire for LGBTQ-specific continuing education, willingness to be listed as an LGBTQ-friendly provider, and belief that the LGBTQ population is more difficult to treat. The practice behaviors section of the survey contained seven statements about practice activities toward LGBTQ patients. These statements examined providers’ habits of inquiring about patients’ sexual orientation, belief in the importance of knowing the sexual orientation and gender identity of patients, belief that being LGBTQ leads to increased cancer risks, assumption of patients’ heterosexuality, feeling well-informed about LGBTQ health needs, and belief there should be mandatory educational events in the workplace on LGBTQ cancer health needs.

The open comments section included four questions that asked about personal experiences treating LGBTQ patients, reservations in treating this population, suggestions for improving the cancer care of this population, and additional comments.

## 3. Statistical Analysis

Descriptive statistics (counts and percentages) were used to quantify survey responses using Stata/MP 12.1 (StataCorp LP, College Station, TX, USA). Open-ended comments were coded using content analysis (Atlas TI) and categorized based on key themes. Two coders initially reviewed all comments to generate a preliminary code list. The initial code list was validated by a third coder and discrepancies were discussed until a consensus was made. The final code list was applied by two coders with an inter-rater reliability of 0.90.

## 4. Results

### 4.1. Demographics

Of the 388 providers contacted, 108 completed the survey, resulting in a 27.8% response rate. For this analysis, we focused on 36 (33.3%) of the 108 respondents who identified as specialists in the seven cancer types with known or suspected LGBTQ-specific disparities [2]: breast, gastrointestinal, genitourinary, gynecological, and thoracic oncology. As presented in Table 1, respondents were primarily male (52.8%), between the ages of 35 and 44 (61.1%), heterosexual (91.7%), non-Hispanic Caucasian (61.1%), Christian (55.6%), and medical doctors (75.0%) who graduated from professional school between 2000 and 2009 (50.0%). Over 44% reported a patient load of between 26 and 50 patients per week, and the majority (72.2%) reported that one to five percent of their patients had identified as LGBTQ in the past year.

### 4.2. Knowledge

Thirteen respondents (36.1%) correctly identified that LGBTQ patients avoid accessing healthcare due to difficulty communicating with healthcare providers (Table 2). 58.3% correctly recognized that HPV-associated cervical dysplasia can be found in lesbians with no history of heterosexual intercourse.

Twenty respondents (55.3%) correctly identified that regularly screening gay and bisexual men for anal cancer through anal Pap testing can increase life expectancy. Seventeen respondents (47.2%) correctly recognized that transgender individuals are less likely to have health insurance than cisgender individuals.

### 4.3. Attitudes

Thirty-three providers (91.7%) stated they were comfortable treating LGBTQ patients (Table 3) and 91.7% believed the LGBTQ population had unique health risks and needs. Twenty-eight (77.8%) agreed there should be more education in health professional schools on LGBTQ health needs. Twenty-three (63.8%) would be willing to be listed as an LGBTQ-friendly provider. Few respondents (four, or 11.1%) believed the LGBTQ population was more difficult to treat.

### 4.4. Practice

Thirteen providers (36.1%) actively inquired about a patient’s sexual orientation when taking a history (Table 4). Twenty-one (58.3%) either agreed or strongly agreed it was important to know the sexual orientation of their patients to provide the best care; only ten providers (27.8%) agreed or strongly agreed with this statement in reference to gender identity. Twenty-six (72.2%) stated that upon first encounter, they assumed a patient was heterosexual. Seventeen (47.2%) agreed or strongly agreed they were well-informed on the health needs of LGBTQ patients. Thirteen (36.1%) agreed or strongly agreed there should be mandatory educational events at the cancer center on LGBTQ health needs.

### 4.5. Open Comments

The open comments section revealed themes related to: disbelief knowing SOGI about patients was important; need for additional education about the importance of knowing and what to do with that information; and lack of knowledge of LGBTQ health needs. The first theme centered on the idea that knowing SOGI was not important in their practice: the majority of providers explained they treat all patients the same regardless of their sexual orientation. For example, the quote below is illustrative of a typical response:

“LGBT patients are no different than heterosexual patients; I treat everyone the same.”

(Thoracic oncology provider)

The second theme centered on the need for provider education and training to understand the importance of knowing a patient’s sexual orientation and gender identity and then how to use that information to provide quality care to patients. These quotes illustrate a typical response:

“I guess I need training as to why this (sexual orientation and gender identity) is important and how I should address it in my practice.”

(Breast oncology provider)

“Obviously I deal with the body parts that relate to sexual acts, but I don’t ask my patients to disclose and I wouldn’t be sure how to apply that information if they did tell me.”

(Gynecologic oncology provider)

Another theme was oncology providers’ lack of knowledge and awareness of LGBTQ cancer-related health issues. The quote below is representative of the lack of knowledge that LGBTQ patients have higher rates of smoking than cisgender and heterosexual populations. The quote from the GI provider shows a lack of knowledge about survivorship and quality of life issues in men who have sex with men and their sexual practices.

“I treat lung cancer patients. Not sure these questions are relevant to my specific area of focus… I imagine GYN and GI oncologist would have differing concerns/areas that need focusing than I.”

(Thoracic oncology provider)

“I see a lot of MSM in my practice but don’t know that knowing their sexual behaviors would change the way I would treat their cancer.”

(Gastrointestinal oncology provider)

## 5. Discussion

In this analysis we assessed knowledge, attitudes, and practice behaviors among a unique sub-group of providers who identified as specialists in one of seven cancer sites that may disproportionately affect LGBTQ populations. Our results suggest all provider groups had poor knowledge of LGBTQ-specific health needs exemplified by less than half of the cancer specialists correctly answering the questions. Additionally, only 47.2% of surveyed providers believed they were well-informed on LGBTQ health needs.

Previous studies have identified similar trends in providers treating LGBTQ patients. For example, prior studies have reported health providers often lack knowledge of LGBT health needs [36,37], and that LGBT patients themselves have to educate their providers on these matters. A survey by Kitt et al. [38] of residents and attending physicians revealed knowledge gaps similar to those found in our study. A report by Stott [39] using interviews and focus groups of general practitioners and LGB individuals, as well as a survey of medical students, also revealed that general practitioners lacked knowledge of the specific health needs of their LGB patients.

With regard to attitudes toward LGBTQ patients, the majority of providers in our study reported they would be willing to be listed as LGBTQ-friendly. As many LGBTQ patients seek LGBTQ-friendly providers, this willingness demonstrates another level of acceptance, as it could potentially increase the number of patients who identify as LGBTQ [40]. A study by VandenLangenberg et al. [41] studied LGB individuals and their genetic counseling experiences through a series of telephone interviews and saw similar results. Specifically, one theme noted that identification as a LGB-friendly provider through LGB-friendly symbols, such as books or stickers, made patients more comfortable with disclosing sexual orientation [41]. LGB-friendly symbols increase patient comfort and foster a welcoming environment. Other ways to make LGB patients feel more comfortable include: providing unisex bathrooms, using gender-neutral intake forms and inclusive language, and LGBTQ-specific posters in waiting rooms [42]. These, along with institutional policies, such as allowing all patients to choose who can visit them in the hospital, opposing “conversion” and other similar therapies for LGBTQ patients [15] may help improve providers’ relationships with LGBTQ patients. Most providers in our study had overall positive attitudes toward the LGBTQ population; however, other studies have found varying results regarding attitudes. Similarly, Lapinski et al. [43] reported that most medical students had positive personal and treatment attitudes toward the LGBTQ population, while other studies have found providers to be less accepting. A recent study by Sabin et al. [44] examined attitudes among healthcare providers toward lesbian women and gay men through the Implicit Association Test [45], which measures the implicit social associations people make between concepts and attributes. The study found heterosexual providers always implicitly preferred heterosexual people over gay and lesbian people. Accordingly, many LGB individuals reported having negative experiences with healthcare providers, often due to perceived providers’ homophobic attitudes [46]. As education and awareness can help providers overcome these implicit biases, this further highlights the need for more education in the area of LGBTQ healthcare.

Oncology providers’ lack of knowledge regarding LGBTQ issues may translate into suboptimal practice behaviors. First, just over half of providers believed it is important to know the sexual orientation or gender identity of patients to provide the best care, and only one-third of surveyed providers actively inquired about sexual orientation when taking a history. However, multiple studies have indicated the importance of knowing patients’ sexual orientation; disclosure has been shown to increase patient satisfaction, improve quality of care, and increase patients’ use of routine care [47,48,49,50]. Other studies have similarly highlighted providers’ lack of inquiry, and have also noted patients are reluctant to disclose sexual orientation on their own [50,51].

While many organizations, including the American Medical Association, recommend providers discuss sexual orientation with patients, our results highlight such guidelines are not always put into practice [48,49]. Other groups and organizations are working to address the cancer burden in the LGBTQ population in various ways—for example, the National Summit on Cancer in the LGBT Communities [52] resulted in recommendations addressing sexual orientation and gender identity data collection, care of LGBT individuals, provider awareness and knowledge, and research on cancer in the LGBT population. The American Cancer Society has created a cancer control program that will work to increase awareness of their resources for the cancer-related needs of the LGBT population and, similar to the National Summit’s Cancer Action Plan, to dedicate research to some of the cancer-related disparities this population faces [53].

The survey’s open comments further highlighted providers lack of knowledge of LGBTQ health needs and how to tailor patient care based on knowing their sexual orientation. Further, the open comment responses suggest latent heteronormativity and do not acknowledge that all patients are individual and as such should be treated according to their specific needs, rather than the same as everyone else. However, the theme of equality also emerged in these comments, as multiple providers explained they treated patients the same way regardless of their sexual orientation. Other studies have found similar results. Specifically, interviews of general practice physicians by Beagan et al. [54] revealed some physicians do not believe sexual or gender identity makes a difference when treating a patient, and that patients should not be labeled by these characteristics. Furthermore, the majority of the Moffitt providers surveyed tend to automatically assume that a patient is heterosexual (72% of respondents). Thus, even when physicians do believe that sexual orientation and gender identity make a difference in caring for a patient, such assumptions can lead providers not to ask questions about sexual orientation and gender identity in the first place. While it is important not to discriminate based on patient sexual orientation or gender identity, it is also important to recognize that LGBTQ individuals have health concerns as the individuals they are and should be treated in a way that respects their identity and individual needs, which may or may not be same as heterosexual and cisgender patients. The provision of quality healthcare is dependent upon not equality, but equity. Specifically, patients should not all be given the same treatment, but quality treatment based on their own individual needs [55,56].

Furthermore, the fact that the vast majority of surveyed providers feel comfortable treating LGBTQ patients, despite knowledge deficits, demonstrates a lack of general awareness of LGBTQ health issues—and therein another potential area of improvement in LGBTQ care. Providers’ support of continuing education/training on LGBTQ-specific cancer related health needs reveals practical ways to improve knowledge of and behaviors toward LGBTQ patients. The Association of American Medical Colleges supports continuing medical education on LGBTQ-related health issues and notes its contribution to an improving providers’ awareness and knowledge of needs, as well as the quality of patient care [57]. Nonetheless, several studies suggest medical education on LGBTQ health is lacking. White et al. [58] found that 67.3% of medical students who took their survey viewed their LGBT-related medical school curriculum as “fair” or worse.

Medical students may be educated on LGBTQ health issues through didactic lectures such as lectures dedicated to LGBTQ health throughout the medical school curriculum, or including issues related to LGBTQ health within the curriculum. Other opportunities include interacting with and interviewing standardized patients who identify as LGBTQ, LGBTQ patient panel discussions, and case studies. A greater focus on medical students’ ability to take a thorough gender identity and sexual and personal history could lessen the barriers physicians report in discomfort in asking for patients’ sexual orientation or gender identity.

We acknowledge several limitations to this study including providers from a single institution, a modest response rate (27.8%), and the low numbers of providers within each of the cancer site specialties. Those who completed the study may have been more familiar with LGBTQ health issues and/or more comfortable with the LGBTQ population than providers who opted not to respond. Clinicians with a more vested interest in LGBTQ cancer health may have been more likely to contribute their time to complete the survey to advance research in this field.

Although this analysis focused on specialists who treat cancers that may disproportionately affect LGBTQ populations, education and training regarding LGBTQ health is important in all medical fields. All oncology providers should be sensitive to and knowledgeable of how to encourage disclosure of sexual orientation and gender identity with their patients.

## Figures and Tables

**Table 1:** Provider demographics in indicated specialties (*N* = 36).

Characteristic ^1^	*N* (%)	Characteristic ^1^	*N* (%)
**Specialty/Moffitt Clinic**		**Religious Identity**	
Breast Oncology	6 (17)	Atheist/Agnostic	1 (3)
Gastrointestinal Oncology	15 (42)	Buddhist	1 (3)
Genitourinary Oncology	4 (11)	Christian	20 (56)
Gynecologic Oncology	4 (11)	Hindu	1 (3)
Thoracic Oncology	7 (19)	Jewish	5 (14)
		Muslim	3 (8)
		Other and Missing	5 (14)
**Gender**		**Licensure**	
Female	17 (47)	MD	27 (75)
Male	19 (53)	PA	4 (11)
		NP	5 (14)
**Age Group**		**Year of Graduation from Professional School**	
25–34	1 (3)	2010–2014	1 (3)
35–44	22 (61)	2000–2009	18 (49)
45–54	9 (25)	1990–1999	14 (39)
55–64	3 (8)	1980–1989	1 (3)
65–74	1 (3)	1970–1979	2 (6)
**Sexual Orientation**		**Average Number of Patients Seen Per Week**	
Heterosexual	33 (97)	0–25	12 (33)
Gay	1 (3)	26–50	16 (44)
		51–75	6 (17)
		75–100	2 (6)
**Race**		**Percentage of Your Patients in the Past Year Who Have Identified Themselves as LGBTQ**	
Asian	5 (14)	None	1 (3)
Black or African American	2 (6)	1–5%	26 (74)
Multiracial	1 (3)	6–10%	5 (14)
Hispanic or Latino	3 (8)	11–15%	1 (3)
White or Caucasian	22 (61)	16–20%	0 (0)
Other/Not sure	3 (8)	>20%	2 (6)

^1^ Not all categories add up to 36 due to missing data.

**Table 2:** Knowledge of LGBTQ health among indicated providers (*N* = 36).

*N* (%) ^1^	
**LGBTQI patients avoid accessing healthcare due to difficulty communicating with providers.**	
Strongly Disagree	5 (13.9)
Disagree	7 (19.5)
Don’t Know	11 (30.5)
Agree Strongly	11 (30.5)
Agree	2 (5.6)
**HPV-associated cervical dysplasia can be found in lesbians with no history of heterosexual intercourse.**	
Strongly Disagree	0 (0.0)
Disagree	5 (13.9)
Don’t Know	10 (27.8)
Agree Strongly	14 (38.9)
Agree	7 (19.4)
**Regularly screening gay and bisexual men for anal cancer through anal Pap testing can increase life expectancy.**	
Strongly Disagree	0 (0.0)
Disagree	2 (5.6)
Don’t Know	14 (38.9)
Agree Strongly	16 (44.4)
Agree	4 (11.1)
**Transgender individuals are less likely to have health insurance than heterosexual individuals.**	
Strongly Disagree	1 (2.8)
Disagree	3 (8.7)
Don’t Know	14 (40.0)
Agree Strongly	16 (45.7)
Agree	1 (2.8)

^1^ Not all responses add up to 36 due to missing data.

**Table 3:** Attitudes regarding LGBTQ patients and healthcare among indicated providers (*N* = 36).

*N* (%) ^1^	
**I am comfortable treating LGBTQ patients.**	
Strongly Disagree	0 (0.0)
Disagree	1 (2.9)
Don’t Know	1 (2.9)
Agree Strongly	13 (37.1)
Agree	20 (57.1)
**The LGBTQ population has unique health risks and needs.**	
Strongly Disagree	0 (0.0)
Disagree	1 (2.9)
Don’t Know	1 (2.9)
Agree Strongly	25 (71.4)
Agree	8 (22.8)
**There should be more education in health professional schools on LGBTQ health needs.**	
Strongly Disagree	1 (2.9)
Disagree	1 (2.9)
Don’t Know	5 (14.2)
Agree Strongly	19 (54.3)
Agree	9 (25.7)
**I would be willing to be listed as an LGBTQ-friendly provider.**	
Strongly Disagree	0 (0.0)
Disagree	2 (5.9)
Don’t Know	9 (26.5)
Agree Strongly	12 (35.3)
Agree	11 (32.3)
**The LGBTQ population is often more difficult to treat.**	
Strongly Disagree	4 (11.4)
Disagree	19 (54.4)
Don’t Know	8 (22.8)
Agree Strongly	4 (11.4)
Agree	0 (0.0)

^1^ Not all responses add up to 36 due to missing data.

**Table 4:** LGBTQ-related practice behaviors among indicated providers (*N* = 36).

*N* (%) ^1^	
**I actively inquire about a patient’s sexual orientation when taking a history.**	
Strongly Disagree	5 (13.9)
Disagree	7 (19.4)
Don’t Know	11 (30.6)
Agree	11 (30.6)
Strongly Agree	2 (5.6)
**It is important to know the sexual orientation of my patients to provide the best care.**	
Strongly Disagree	0 (0.0)
Disagree	5 (13.9)
Don’t Know	10 (27.8)
Agree	14 (38.9)
Strongly Agree	7 (19.4)
**It is important to know the gender identity of my patients to provide the best care.**	
Strongly Disagree	3 (8.3)
Disagree	10 (27.8)
Don’t Know	13 (36.1)
Agree	9 (25.0)
Strongly Agree	1 (2.8)
**Upon first encounter I assume a patient is heterosexual.**	
Strongly Disagree	1 (2.8)
Disagree	0 (0.0)
Don’t Know	9 (25.0)
Agree	19 (52.8)
Strongly Agree	7 (19.4)
**I am well informed on the health needs of LGBTQ patients.**	
Strongly Disagree	1 (2.8)
Disagree	3 (8.7)
Don’t Know	14 (40.0)
Agree	16 (45.7)
Strongly Agree	1 (2.8)
**There should be mandatory educational events at Moffitt on LGBTQ health needs.**	
Strongly Disagree	5 (13.9)
Disagree	7 (19.4)
Don’t Know	11 (30.6)
Agree	11 (30.6)
Strongly Agree	2 (5.6)

^1^ Not all responses add up to 36 due to missing data.
